# The hemodynamics of adrenal veins with four-dimensional computed tomography using quantitative time-density curve: a study based on aldosteronism patients

**DOI:** 10.1038/s41598-023-41414-9

**Published:** 2023-09-01

**Authors:** Xi He, Eijun Sueyoshi, Hiroki Nagayama, Hirofumi Koike, Masataka Uetani

**Affiliations:** 1 Department of Radiological Sciences, Graduate School of Biomedical Sciences, Nagasaki University, 1-7-1 Sakamoto, Nagasaki, 852-8521 Japan; 2 Department of Radiology, Nagasaki Harbor Medical Center, Nagasaki, Japan

**Keywords:** Diseases, Endocrinology, Urology

## Abstract

Present study quantitatively analyzed adrenal venous flow using four-dimensional computed tomography (4D CT). We reviewed 4D CT images of 55 patients [mean age, 52 years ± 11 (standard deviation); 23 females] who underwent adrenal venous sampling between August 2017 and February 2021. Time–density curves were referred for the adrenal venous enhancement. The clinical factors affecting hemodynamics were assessed using uni- and multivariate linear regression analyses. The right and left adrenal veins (RAV and LAV, respectively) were visualized in all cases. Mean peak enhancement values in RAV and LAV were 247 ± 67 and 292 ± 70 Hounsfield units (*P* < 0.01), and were reached at 44.43 ± 6.86 and 45.39 ± 7.53 s (*P* < 0.01), respectively. The body mass index (BMI), plasma renin activity and potassium were significant factors influencing the peak enhancement of RAV blood flow [standardized regression coefficients, − 0.327 (*P* = 0.017), − 0.346 (*P* = 0.013), 0.426 (*P* = 0.016), respectively]. A linear relationship between sex and the time-to-peak was observed for RAV [standardized regression coefficient, 0.348 (*P* = 0.046)]. RAV had a lower contrast effect than LAV and reached its peak faster. BMI, plasma renin activity, and potassium were associated with flow density in RAV. Sex independently influenced the time-to-peak.

## Introduction

The measurement of aldosterone and cortisol levels in the adrenal veins using adrenal venous sampling (AVS) is the gold standard for identifying the subtypes of primary aldosteronism^1^. Although AVS is a reliable reference, it is an invasive and technically challenging procedure mainly because blood sampling from the right adrenal vein (RAV) is small and susceptible to anatomical variations. Previous studies demonstrated the accuracy and validity of imaging protocols, including dynamic CT, for the pre-interventional mapping of sampling veins, which may facilitate successful AVS; however, the delineation rate of RAV is not always high^2–4^, and limited information is currently available on the hemodynamics of the adrenal veins.

Although previous study has reported the utility of 4D CT for evaluating hemodynamic characteristics in hepatocellular carcinoma and intracranial veins, the application is still limited due to the insufficient temporal resolution^5, 6^. According to the effective method proposed for the whole brain 4D CT angiography before, we enhanced the temporal resolution and clarified the adrenal venous flow dynamics by analyzing the time-density curve (TDC) of adrenal veins^6^.

The present study quantitatively investigated adrenal venous flow using data derived from 4D CT, including the attenuation maximum and time-to-peak parameter, which measures the duration before TDC peaks. Semi-quantitative characteristics, such as the slope of TDC, and the clinical factors that affect hemodynamics were also examined.

## Materials and methods

### Patient collection

The present study was approved by the Institutional Review Board of Nagasaki University Hospital (No. 21081608). This research was performed in accordance with the relevant guidelines and due to the retrospective study design, the Ethics Committee of the Nagasaki University Hospital provided a waiver for informed consent of the study. Research involving human research participants must have been performed in accordance with the Declaration of Helsinki. The 4D CT scans of consecutive patients with primary aldosteronism, selected from our institution between August 2017 and February 2021 were performed according to the guidelines published by the Japan Endocrine Society^7^. And the images obtained were independently reviewed by two blinded readers. Patients with Cushing’s syndrome or receiving steroid treatment for contrast allergy were excluded because their cortisol concentrations were unable to be used for sampling. Detailed serological data and basic information were recorded. The preliminary suspicion of primary aldosteronismwas based on arterial hypertension, and an increased aldosterone-to-renin ratio and serum aldosterone > 137 pM after the infusion of 2 L of 0.9% saline in a suppression test confirmed the diagnosis. Unilateral excessive aldosterone secretion was diagnosed when the aldosterone-to-cortisol ratio from one adrenal vein was ≥ fourfold higher than that of the contralateral side^7^.

### CT examination

Contrast-enhanced 4D CT was performed within seven days prior to AVS using a 320-row detector scanner (Aquilion One; Canon Medical Systems). Scanning parameters were as follows: 11 phases with the volume scan mode; 100 kVp with Real EC, 150 mA tube current, and 0.5 s per rotation; 0.5 mm × 320 collimation and CTDI_vol_ of 54.7 mGy. All settings were as consistent as possible among patients. A non-ionic contrast bolus (Imeron, Bracco Imaging Deutschland GmbH, Konstanz, Germany) was injected into the antecubital vein with 600 mg of iodine per kilogram of body weight. The injection time was fixed at 25 s, achieving a varied injection rate in accordance with patient’s weight. Images were continuously obtained in 11 series at 3-s intervals, beginning from 25 s after the onset of the contrast injection during the same breath hold in the shallow expiratory phase. A few patients failed to hold their breath at the last one or two phases, but the optimal visibility of venous vessels was still obtained. According to the range confirmed by pre-contrast abdominal scan, the imaging area was 160 mm centered on the right adrenal gland^8^. The reconstruction parameters were as follows: FC 13 kernel, 0.5 mm slice thickness with intervals of 0.25 mm. As for the exposure, we made 4D CT scanning within the radiation dose of 3D CT protocol.

### CT interpretation

CT images were evaluated by two diagnostic radiologists. One of them has 20 years of working experience as a radiologist and the other has been working for 15 years in radiological sciences. They performed the image review and measurements independently. When the results were different, the selection and placement of regions of interest (ROI) would be performed through consensus by the two reviewers. Each phase was evaluated on a workstation using Vincent software (Fujifilm Medical, Tokyo, Japan) when the optimized visualization of the bilateral adrenal veins was selected.

**Figure 1:**
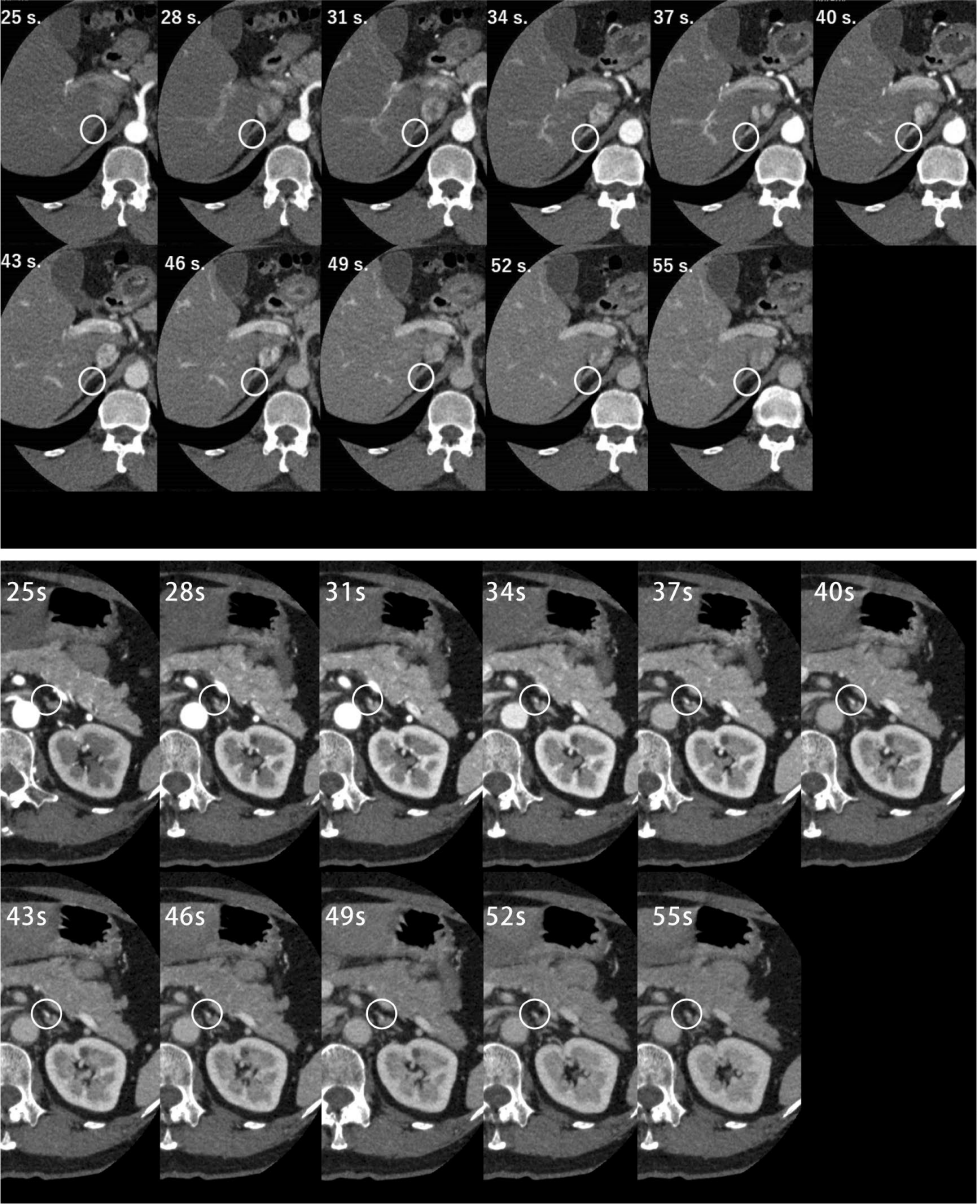
Attenuation values of adrenal veins were measured at 11 phases in a set of ROIs over the straight portion.

The attenuation values of the adrenal veins were measured at 11 phases in a set of ROIs over the straight portion. The central adrenal veins were measured in both the left and right sides (Fig. 1). The projection of streak and beam-hardening artifacts was carefully avoided. The ROI density values of each vein were plotted as curves versus time using GraphPad Prism 7. First-order relative differences (%) between continuous observations were calculated to obtain the peak enhancement [D_max_ in Hounsfield units (HU)] and the time to peak [t_max_ in seconds], at which the contrast concentration reached the maximum level. These hemodynamic values were obtained when the relative difference changed from positive to negative. In the case of a plateau density with possible measurement errors, peak enhancement values were retrieved when the relative difference suddenly declined below the threshold of 10%. The downward slope of TDC was also calculated.

According to the pre-mapping, the positions of left adrenal vein (LAV) and RAV on CT and catheter venogram were compared. The presence or absence of adrenal nodules was independently assessed by the two radiologists, and a decision was made by consensus for cases where they differed. We defined patients with adrenal nodules as those with nodules (or thickening) of at least 10 mm detected at staging CT. We referred to the 10-mm threshold because many guidelines recommend a work-up for adrenal nodules that are 1 cm or larger, whereas a threshold less than 10 mm may lead to false-positive results^9^.

### Clinical indexes

The database was reviewed to record the following variables: (I) general information, including sex, age, body mass index (BMI), and smoking and drinking history; (II) pre-interventional observation indexes, including the location and number of adrenal nodules detected by 4D CT, systolic and diastolic blood pressure values; (III) diagnostic results of AVS; and (IV) preoperative hematological data, including serum creatinine, plasma aldosterone concentrations, plasma renin activity, and sodium and potassium levels.

### Statistical analysis

Demographic and clinical data were analyzed using IBM SPSS Statistics (version 22.0, Chicago, IL, USA). Continuous variables were expressed as the mean ± standard deviation, and categorical data were summarized in terms of counts with corresponding percentage in parentheses. The Student’s *t*-tests were performed for paired data and the Wilcoxon signed-rank test was applied when data did not show a normal distribution. Categorical variables were analyzed using Fisher’s exact test. Additionally, the mean values of each quantitative parameter from bilateral density response curves were calculated (D_max_, t_max_, and slope) and compared by a one-way analysis of variance based on the side. Uni- and multivariate analyses were conducted on the whole cohort to identify relationships between clinical factors and D_max_ and t_max_, including the following parameters as covariates: sex (male/female); age (as a continuous variable); adrenal nodules (none, left, right, or both sides); and continuous serological data: BMI, serum creatinine, plasma aldosterone concentrations, plasma renin activity, sodium and potassium levels, and systolic and diastolic blood pressure. Unstandardized coefficients [β, with 95% confidence intervals (95% CIs)] and standardized coefficients (β) were recorded. All *P*-values were two-sided and those less than 0.05 in the multivariate analysis were considered significant.

## Results

### Patient sample

Screening out those who had Cushing’s syndrome (n = 8) and who received steroid treatment (n = 3), fifty-five patients with primary aldosteronism following the guidelines published by the Japan Endocrine Society were included and their demographic data are listed in Table 1^7^. The mean age of patients was 52 ± 11 years (range, 41–63 years), and 32 were males (58.2%). The adrenal glands in the whole cohort were examined (n = 110). Of which, twenty-nine cases presented with adrenal nodules (14.5% right, 25.5% left, and 12.7% both sides). Aldosterone-producing lateralization was considered right, left, and bilateral secretion in 23.6, 29.1, and 47.3% of patients, respectively. In all cases, the positions of RAV and the LAV were consistent with the results of AVS. The RAV location was considered the 11th thoracic vertebra, the 12nd thoracic vertebra and the 1st lumbar vertebra, respectively. Thirty patients had the confluence of RAV and accessory hepatic vein. All the LAV drained into the left renal vein and they were located in the 12nd thoracic vertebra, the 1st lumbar vertebra and the 2nd lumbar vertebra, respectively.

**Table 1:** Patient characteristics.

Characteristic	All cases (n = 55)
Gender^b^	
Male	32 (58.2)
Female	23 (41.8)
Age (y)^a^	52 ± 11
Adrenal nodules^b^	
None	26 (47.3)
Right side	8 (14.5)
Left side	14 (25.5)
Both sides	7 (12.7)
PA subtypes^b,c^	
Right	13 (23.6%)
Left	16 (29.1%)
Bilateral	26 (47.3%)
Smoking^b^	
Yes	26 (47.3%)
No	29 (52.7%)
Drinking^b^	
Yes	22 (40%)
No	33 (60%)
BMI (kg/m^2^)^a^	26 ± 4
Serum creatinine(mg/dL)^a^	0.75 ± 0.16
PAC (pg/ml)^a^	217.17 ± 158.37
PRA (ng/ml/h) ^a^	0.36 ± 0.22
Sodium (mEq/L)^a^	141.33 ± 1.60
Potassium (mEq/L)^a^	3.43 ± 0.41
Systolic BP (mmHg)^a^	138 ± 16
Diastolic BP (mmHg)^a^	88 ± 15

*PA* primary aldosteronism, *BMI* body mass index, calculated by taking a person’s weight, in kilograms, divided by their height, in meters squared, *PAC* plasma aldosterone concentrations, *PRA* plasma renin activity, *BP* blood pressure.

^a^Continuous variables are reported as mean ± standard deviation or median and interquartile range.

^b^Categorical variables are reported as number, with the corresponding percentage in parentheses.

^c^Guidelines for the diagnosis and treatment of primary aldosteronism–the Japan Endocrine Society 2009.

*P < 0.05.

### Flow dynamics

**Figure 2:**
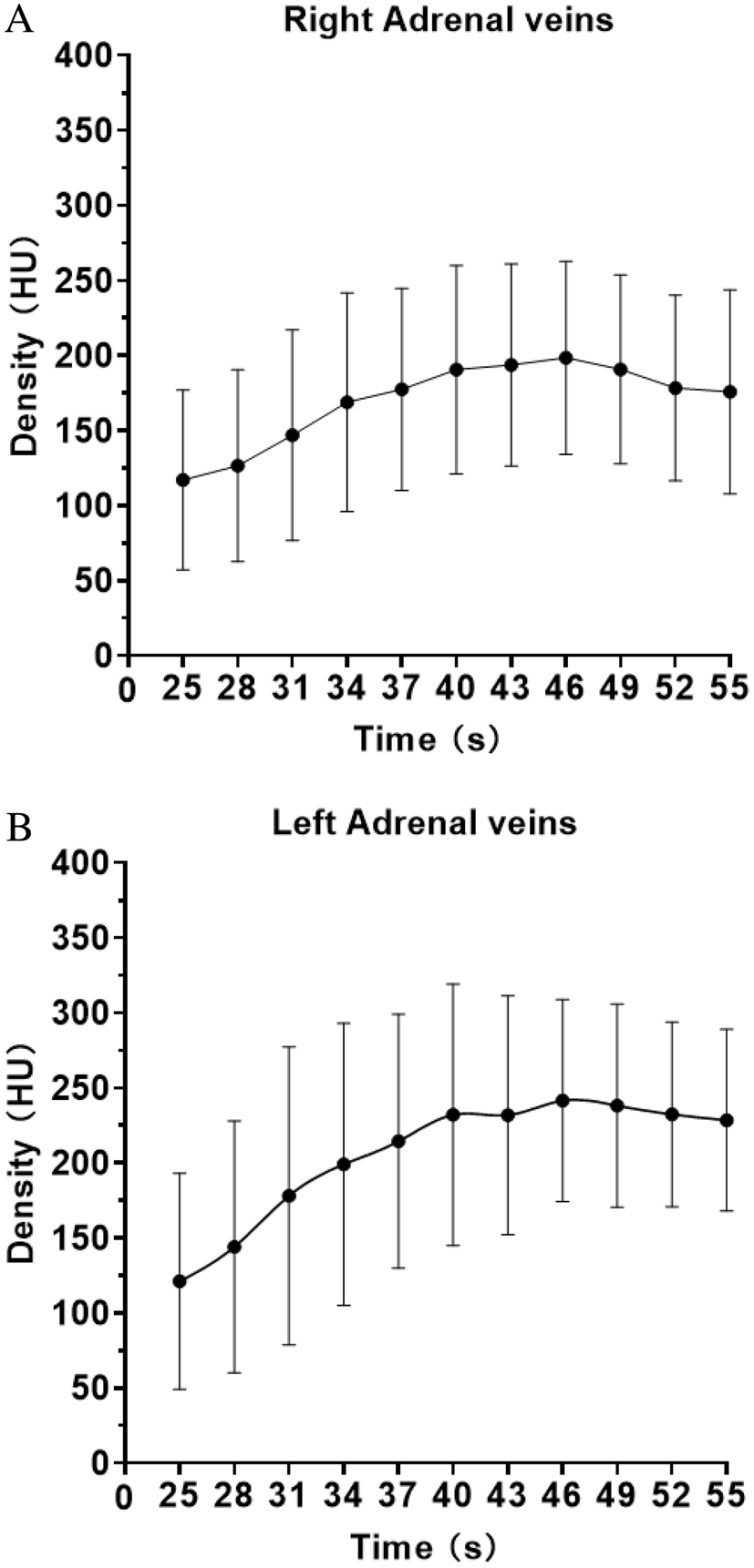
(**A**) In RAV, D_max_ was 246.91 ± 67.37 HU and t_max_ was 44.43 ± 6.86 s. (**B**) In LAV, D_max_ was 291.57 ± 70.00HU and t_max_ was 45.39 ± 7.53 s. Therefore, the optimal time window for 4D CT was obtained between 36 and 54 s in both RAV and LAV.

In conventional contrast-enhanced CT examination, the RAV is sometimes not identified while in this study, the right and left adrenal veins were all recognized. Overall, 110 veins (100%) were examined and TDC are shown in Fig. 2A,B. Mean D_max_ and t_max_ for RAV and LAV are shown in Table 2. D_max_ significantly differed between RAV and LAV (247 ± 67 vs. 292 ± 70 HU, *P* < 0.01), and were reached at 44.43 ± 6.86 and 45.39 ± 7.53 s, respectively (*P* < 0.01). LAV exhibited significantly higher mean peak enhancement than RAV. The downward slope of the RAV curve was less steep than that of LAV (1.96 ± 3.76 vs. 3.58 ± 4.54 HU, *P* < 0.01).

**Table 2:** Peak enhancement (D_max_) and time to reach peak enhancement (t_max_) in adrenal veins.

Venous side	Right	Left	*P* value
D_max_ (HU)	247 ± 67	292 ± 70	< 0.01*
t_max_ (s)	44.43 ± 6.86	45.39 ± 7.53	< 0.01*
Slope^†^	1.96 ± 3.76	3.58 ± 4.54	< 0.01*

*P < 0.05.

Values reported are mean standard ± deviation.

^†^The downward slope of the time-density curve.

### Univariate and multivariate analyses of clinical factors influencing D_max_ based on 4D CT

In Table 3, the univariate analysis revealed that only the D_max_ of RAV was affected by clinical factors, including BMI [Unstandardized Regression Coefficient (UC) = − 5.211, Standardized Regression Coefficient (SC) = − 0.319, *P* = 0.019] and plasma renin activity (UC = − 85.591, SC = − 0.299, *P* = 0.028). No significant differences were observed in potassium levels between RAV and LAV peak enhancement (*P* = 0.181). BMI, plasma renin activity, and potassium levels independently correlated with D_max_ in RAV in the multivariate analysis (*P* = 0.017, 0.013, and 0.016, respectively).

**Table 3:** Uni- and multivariate linear regression analysis of clinical factors for peak enhancement (D_max_) based on 4D CT.

Variables^†^	Left						Right					
	Univariate analysis			Multivariate analysis			Univariate analysis			Multivariate analysis		
	Unstandardized coefficients	Standardized coefficients	*P* value	Unstandardized coefficients	Standardized coefficients	*P* value	Unstandardized coefficients	Standardized coefficients	*P* value	Unstandardized coefficients	Standardized coefficients	*P* value
Gender	− 4.655	− 0.034	0.808	15.437	0.112	0.499	− 17.543	− 0.141	0.311	− 23.138	− 0.185	0.237
Age (years)	0.724	0.112	0.420	0.291	0.045	0.778	0.948	0.167	0.228	0.574	0.101	0.525
Adrenal nodules	16.374	0.268	0.050	19.479	0.319	0.063	13.804	0.249	0.069	7.760	0.140	0.319
BMI (kg/m^2^)	− 1.197	− 0.067	0.633	− 0.691	− 0.038	0.797	− 5.211	− 0.319	0.019*	− 5.346	− 0.327	0.017*
Serum creatinine (mg/dL)	− 57.841	− 0.136	0.328	− 84.434	− 0.198	0.257	− 11.562	− 0.03	0.83	− 6.832	− 0.018	0.917
PAC (pg/ml)	0.002	0.006	0.968	0.013	0.030	0.856	− 0.048	− 0.123	0.377	0.031	0.078	0.589
PRA (ng/ml/h)	− 71.318	− 0.226	0.100	− 48.179	− 0.153	0.314	− 85.591	− 0.299	0.028*	− 99.148	− 0.346	0.013*
Sodium (mEq/L)	5.770	0.135	0.331	4.592	0.107	0.540	1.586	0.041	0.770	6.558	0.169	0.284
Potassium (mEq/L)	− 25.657	− 0.154	0.267	0.536	0.003	0.987	28.060	0.185	0.181	64.687	0.426	0.016*
Systolic BP (mmHg)	− 0.673	− 1.154	0.254	− 0.265	− 0.062	0.770	0.265	− 0.068	0.623	0.324	0.084	0.647
Diastolic BP (mmHg)	− 0.919	− 0.202	0.143	− 0.212	− 0.047	0.830	0.712	− 0.172	0.213	− 0.284	− 0.069	0.723

*BMI* body mass index, calculated by taking a person’s weight, in kilograms, divided by their height, in meters squared, *PAC* plasma aldosterone concentrations, *PRA* plasma renin activity, *BP* blood pressure.

*P < 0.05.

^†^Data are β values, and the data in parentheses are 95% confidence interval.

### Univariate and multivariate analyses of clinical factors influencing t_max_ based on 4D CT

A linear regression analysis of LAV and RAV was also performed to identify factors associated with time-to-peak enhancement values, and the results obtained are shown in Table 4. In the univariate analysis, plasma aldosterone concentrations significantly affected t_max_ in RAV and LAV (*P* = 0.043 and 0.048, respectively), whereas systolic blood pressure only affected t_max_ in LAV (*P* = 0.010). Sex positively correlated with t_max_ in RAV in the univariate and multivariate analyses (SC = 0.282 and 0.348, *P* = 0.039 and 0.046, respectively).

**Table 4:** Uni- and multivariate linear regression analysis of clinical factors for time-to-peak parameter (t_max_) based on 4D CT.

Variables^†^	Left						Right					
	Univariate analysis			Multivariate analysis			Univariate analysis			Multivariate analysis		
	Unstandardized coefficients*	Standardized coefficients*	*P* value	Unstandardized coefficients	Standardized coefficients	*P* value	Unstandardized coefficients	Standardized coefficients	*P* value	Unstandardized coefficients	Standardized coefficients	*P* value
Gender	0.951	0.062	0.654	− 0.600	− 0.039	0.809	3.791	0.282	0.039*	4.681	0.348	0.046*
Age (years)	0.048	0.067	0.633	− 0.021	− 0.030	0.850	0.031	0.050	0.719	− 0.037	− 0.060	0.731
Adrenal nodules	− 0.524	− 0.077	0.578	− 0.427	− 0.063	0.703	− 0.054	− 0.009	0.948	0.133	0.022	0.884
BMI (kg/m^2^)	− 0.213	− 0.107	0.443	− 0.169	− 0.085	0.564	− 0.187	− 0.106	0.444	− 0.185	− 0.105	0.466
Serum creatinine (mg/dL)	4.326	0.091	0.511	6.398	0.135	0.430	4.699	0.113	0.417	1.019	0.024	0.896
PAC (pg/ml)	0.013	0.276	0.043*	0.012	0.253	0.120	0.011	0.271	0.048*	0.006	0.148	0.352
PRA (ng/ml/h)	− 0.709	− 0.020	0.884	− 0.900	− 0.026	0.862	− 1.188	− 0.039	0.782	− 1.132	− 0.037	0.803
Sodium (mEq/L)	− 0.191	− 0.040	0.773	− 0.156	− 0.033	0.848	− 0.015	− 0.004	0.980	− 0.388	− 0.093	0.589
Potassium (mEq/L)	0.058	0.003	0.982	1.605	0.087	0.644	− 2.057	− 0.126	0.364	− 3.205	− 0.196	0.296
Systolic BP (mmHg)	− 0.165	− 0.349	0.010*	− 0.167	0.353	0.097	− 0.109	− 0.263	0.055	− 0.107	− 0.256	0.203
Diastolic BP (mmHg)	− 0.092	− 0.181	0.190	0.013	0.026	0.903	− 0.055	− 0.123	0.376	− 0.003	− 0.007	0.972

*BMI* body mass index, calculated by taking a person’s weight, in kilograms, divided by their height, in meters squared, *PAC* plasma aldosterone concentrations, *PRA* plasma renin activity, *BP* blood pressure.

*P < 0.05.

^†^Data are β values, and the data in parentheses are 95% confidence interval.

## Discussion

Although AVS is a well-established technique, the development of a more convenient and simple method is desired since the reported success rate of RAV catheterization is as low as 70%^10^. Multi-row detector CT and magnetic resonance imaging (MRI) are commonly used in the preoperative delineation of the adrenal veins; however, there is still a degree of inadequacy in the detection rate of RAV^6–8, 16^. Although MRI is effective in avoiding the radiation exposure or complications resulting from contrast agent, CT has acquired detectability of RAV than non-contrast-enhanced MR techniques such as 4D-flow MRI^3^. Because it requires a special sequence, which may be unavailable in some hospitals, and the process for obtaining images is long. Another reason to employ 4D CT is the sufficient image quality, in which submillimeter resolution is available. Also, existing study shows the difficulty in quantifying blood flow reliably through MRI acquisitions from irregular cardiac cycles^11^. Additionally, the scan delay for the optimal visualization of RAV remains controversial. Ota et al.^7^ reported that obtaining the late arterial phase 13 s after the first scan allowed the RAV to be delineated in 93% of cases. Degenhart et al.^2^ performed scanning at a 90-s delay, yielding a rate of only 70–88% in the late portal phase. However, Morita et al.^12^ indicated that it was possible to visualize RAV in the single venous phase and, thus, dual adrenal venous images were used, which corresponded to the period between the portal venous phase and late arterial phase. Delays of 45 and 55 s were applied and the RAV visualization rate increased to 98%. In the present study, the optimal time window for 4D CT was between 36 and 54 s in RAV and LAV. Besides, bilateral adrenal veins were detected in all patients on images and the extraction of flow dynamic features enabled us to easily evaluate enhancement patterns over time, which is more feasible than the above protocols.

A higher CT value was achieved for RAV than previously reported in each phase of less than 200 HU^12^. Our results also showed that right and left t_max_ were 44.43 ± 6.86 and 45.39 ± 7.53 s, respectively. If the injection method of contrast medium is the same, even in facilities without 4D CT, RAV and LAV may be clearly visualized if the protocol is designed to take images including 44 and 45 s after the start of the injection. In this study, we improved the temporal resolution and clarified the adrenal venous flow dynamics by analyzing the TDC of adrenal veins according to previous literature, this enabled us to delineate the RAV as well as the best scan times for 4D CT, which may improve the AVS benefits^6^. Our results could enhance the knowledge of venous physiology and pathophysiology. In addition to the complex anatomical lesions, rapid flow empties from adrenal veins to the confluence veins may cause difficulty in AVS, for which understanding the venous flow dynamics is fundamentally important.

Another study demonstrated that TDC was effective in facilitating the evaluation of contrast wash-in and wash-out phases, allowing for superior visualization of blood vessels^11^. Respiration-induced motion artifacts may affect the evaluation of small regions, particularly when the effect is present in the single arterial phase. Therefore, covert variations in RAV and LAV may have been overlooked because enhancement differences were previously reported to be significant in the affected series^13^. Since multiple phases were available in the present study, movement artifacts were less problematic.

A significant difference was observed in D_max_ between RAV and LAV, with RAV having a lower value. This was mainly due to the short length of RAV and its diameter of < 2–3 mm, which generally does not allow for satisfactory anatomical mapping. Furthermore, the drainage of the central vein on the right side may have been duplicated, which presumably led to a change in the concentration of the contrast agent^8^.

In the present study, the t_max_ of RAV and LAV reached enhancement earlier in RAV. Since the confluence of the adrenal central vein and inferior vena cava is common, the short t_max_ of RAV may be related to fast outflow from the renal vein to the adrenal vein^20^. LAV is defined as the entirety of the left adrenal central vein up to its connection with the left renal vein. A previous study also identified an abnormal confluence of LAV with the periaortic left renal vein and the direct integration of LAV with the inferior vena cava^18^. Therefore, outcomes may differ if the LAV measurement position is above the confluence of the renal veins or upstream of the confluence of the inferior transverse veins. In addition, the flow of LAV may be affected by recirculation, resulting in a longer t_max_.

The multivariate analysis showed that BMI, plasma renin activity, and potassium significantly affected the 4D CT image intensity of blood flow in RAV. This finding suggests that BMI alterations specific to the peak enhancement may allow for the personalization of RAV delineation^14^. In addition, previous studies suggested that contrast medium concentration varies with plasma volume and extracellular fluid volume in parenchymal organs, and that lean body mass and fluid retention, which are associated with BMI, can affect venous enhancement^21^. The expansion and distribution of extracellular fluids beside RAV may differ from that around LAV because of the closely adjacent liver parenchyma, and the TDC of the liver was previously shown to be similar to that of RAV^14, 15^. On the other hand, the contrast materials administered in the blood compartment may be diluted less in RAV than in LAV due to its small size, resulting in a lower concentration in blood flowing in RAV^16^. These parameters need to be considered when performing a dynamic analysis of RAV to avoid over- or underestimations of enhancement and contrast concentrations in veins.

Elevated aldosterone with suppressed plasma renin activity is a characteristic finding of primary aldosteronism^17^. However, few studies have reported a correlation between blood flow and plasma renin activity, and the usefulness of plasma renin activity as a reference marker to evaluate RAV has not yet been examined^18^. Experimental observations showed that renovascular hypertension was mainly maintained by the renin–angiotensin–aldosterone system (RAAS). Volume retention increased blood pressure, and fluid expansion returned negative feedback to the RAAS, leading to a higher plasma renin activity level than expected with volume overload. High plasma renin activity may activate calcium mobilization and vasoconstrictor effect may result in renal artery stenosis^19^. In the present study, data from RAV suggested a peak enhancement change with plasma renin activity, which may have resulted from venous blood volume and vascular lumen sizes influencing blood flow^19, 20^.

The classical paradigm indicates that retained excess sodium is excreted, thereby returning the extracellular fluid volume excess to a normal level and deactivating negative feedback on the RAAS^19^. While this study showed that the contrast medium concentration in the adrenal vein was not correlated with the sodium concentration, and the potassium concentration significantly affected the 4D CT image intensity of blood flow in the RAV. Baudrand et al.^17^ reported that urinary potassium excretion was higher than sodium excretion and did not stimulate renin; however, our results showed that the stimulatory effects of potassium on blood flow were stronger than those of sodium. The reason for this discrepancy remains unclear and may be due to the lack of confounding factors controlling RAAS in the present study, such as antihypertensive medication and the dietary intake of potassium and sodium; therefore, further studies are warranted.

A linear relationship between sex and the time-to-peak parameter was observed in RAV. Although previous studies demonstrated that sex strongly correlated with sustained hypertension in primary aldosteronism patients, the relationship between adrenal vein hemodynamics and sex remains unknown. The optimal time window for delineating bilateral adrenal veins in our protocol was the same; however, a precise time still needs to be considered for taking advantage of optimal venous enhancement.

There were some limitations that need to be addressed. The present study was retrospective, performed at a single center, and included a small sample with potential selective bias. Thus, more in-depth statistical analysis and multicenter study are necessary to eliminate bias and to establish the undisputed value of hemodynamics extracted from 4D CT for delineating adrenal veins. Furthermore, the present results were based only on imaging features without sub-analyses after dividing by the anatomical patterns, and all these interpretations may not be consistent with the anatomy of patients^2^. Moreover, we were unable to confirm the performance of images because they were obtained with contrast agents and were influenced by the protocol and baseline characteristics of patients. Another limitation is that ROI was placed manually with possible measurement errors, which may be avoided with automated selection in the future. In addition, the non-uniform distribution of contrast agent, pulsation, continuous contrast agent diffusion, and blood turbulence may have had a considerable impact. These limitations cannot be ignored when interpreting the present results and, thus, further studies are required.

In conclusion, a lower contrast effect and earlier peak enhancement was achieved for blood flow in RAV than in LAV. BMI, plasma renin activity, and potassium positively correlated with the blood flow density in RAV, and sex was independently associated with the time at which maximum CT attenuation values of RAV were obtained.

## Data Availability

The datasets used and/or analysed during the current study available from the corresponding author on reasonable request.
